# How Do Humans Perform in Multiple Object Tracking With Unstable Features

**DOI:** 10.3389/fpsyg.2020.01940

**Published:** 2020-07-31

**Authors:** Chen Zhao, Luming Hu, Liuqing Wei, Chundi Wang, Xiaowei Li, Bin Hu, Xuemin Zhang

**Affiliations:** ^1^Faculty of Psychology, Beijing Normal University, Beijing, China; ^2^Department of Psychology, Institute of Education, Hubei University, Wuhan, China; ^3^Department of Psychology and Research Centre of Aeronautic Psychology and Behavior, Beihang University, Beijing, China; ^4^Gansu Provincial Key Laboratory of Wearable Computing, School of Information Science and Engineering, Lanzhou University, Lanzhou, China; ^5^CAS Center for Excellence in Brain Science and Intelligence Technology, Shanghai Institutes for Biological Sciences, Chinese Academy of Sciences, Shanghai, China; ^6^Beijing Institute for Brain Disorders, Capital Medical University, Beijing, China; ^7^Faculty of Psychology, State Key Laboratory of Cognitive Neuroscience and Learning, Beijing Normal University, Beijing, China; ^8^Beijing Key Laboratory of Applied Experimental Psychology, National Demonstration Center for Experimental Psychology Education, Faculty of Psychology, Beijing Normal University, Beijing, China

**Keywords:** multiple object tracking, feature changing, feature heterogeneity, working memory updating, feature utilization

## Abstract

In real-world scenarios, objects’ surface features sometimes change as they move, impairing the continuity of objects. However, it is still unknown how our visual system adapts to this dynamic change. Hence, the present study investigated the role of feature changes in attentive tracking through a modified multiple object tracking (MOT) task. The feature heterogeneity and feature stability were manipulated in two experiments. The results from Experiment 1 showed that the tracking performance under feature-changed condition was lower than that under the feature-fixed condition only when the objects were four colors grouped or all unique, suggesting that the performance decrease was moderated by the feature heterogeneity. In Experiment 2, we further examined this effect by manipulating the frequency of feature change. The results showed that when the target set was one color or two colors grouped (the color grouping for the distractor set corresponded with it), the tracking performance decreased significantly as the feature-change frequency increased. However, this trend was not the case when the objects were of the same color or eight unique colors. In addition, a relatively consistent effect appeared both in Experiments 1 and 2. When objects have unique features, the tracking performance decreased significantly as the increase of feature heterogeneity in each frequency of feature changes. Taken together, we concluded that unstable features could be utilized in attentive tracking, and the extent to which the observers relied on surface feature information to assist tracking depended on the level of feature heterogeneity and the frequency of feature change.

## Introduction

How humans assign visual attentional resources in multifocal dynamic scenes has typically been studied using the multiple object tracking (MOT) task (Pylyshyn and Storm, 1988). In the MOT task, observers are required to attend and track a subset of predefined targets among identical distractors. Previous studies showed that the task of tracking moving objects was actively attention-demanding (Kunar et al., 2008; Tombu and Seiffert, 2011; Meyerhoff et al., 2017). Moreover, objects’ spatiotemporal features rather than their surface features usually occupied most of the attentional resources in this task (Pylyshyn, 2004; Feldman and Tremoulet, 2006; Mitroff and Alvarez, 2007). Objects’ surface features seemed to be poorly retained in attentive tracking and had little effect on the establishment and maintenance of object continuity. However, these studies might have underestimated the importance of objects’ features, since the objects in these experiments were all identical, which made it helpless for the observers to use them to assist with tracking (Makovski and Jiang, 2009).

Recently, many studies have demonstrated that objects’ surface features could affect attentive tracking in different ways (Horowitz et al., 2007; Howard and Holcombe, 2008; Makovski and Jiang, 2009; Erlikhman et al., 2013; Wang et al., 2016, 2019; Meyerhoff et al., 2017). First, objects’ unique features could facilitate attentive tracking. Using uniquely-colored objects as stimuli, Makovski and Jiang (2009) found that the tracking performance was enhanced in the unique condition (i.e., eight objects in eight different colors) comparing to that in the homogeneous condition (i.e., eight objects of the same color). Second, the feature distinctiveness between targets and distractors could also assist in attentive tracking. Feria (2012) indicated that distractors that were distinct from the targets in specific properties were more likely to assist with attentive tracking than those that shared identical properties with the targets. Erlikhman et al. (2013) showed that when targets were grouped automatically based on the same feature, the tracking performance was significantly improved. When targets were grouped with distractors automatically based on the same feature, however, the tracking performance was significantly impaired. Wang et al. (2019) further demonstrated that the tracking performance could be affected by different levels of feature distinctiveness between the target and distractor sets. Finally, the feature complexity of the objects could affect attentive tracking. Wang et al. (2019) manipulated the number of colors to define the feature complexity of the target set and the distractor set. They found that only the feature complexity of the target set rather than of the distractor set could significantly affect tracking performance. Taken together, these results provided evidence that surface features could be used for attentive tracking, and the variation of surface features such as uniqueness, distinctiveness, and complexity had a strong effect on attentive tracking.

However, almost all of the available studies have typically focused on the role of stable features (i.e., the feature is unchanged). There is still a vital yet largely neglected issue: to what extent do unstable features affect attentive tracking? In other words, if the feature changes during motion, would the observers’ tracking performance be impaired or not? In real-world tracking scenarios, features can change as an object moves. Therefore, studies of feature changes could help to reach a better understanding of how unstable features play a role in real-world attentive tracking. Previous studies provided evidence that the stable features of objects were coded and stored in visual working memory (VWM) during multiple objects attentive tracking (Makovski and Jiang, 2009; Meyerhoff et al., 2017; Wang et al., 2019). The exploration of unstable features, therefore, inevitably involves the function of VWM maintenance. More importantly, it also involves the function of VWM refresh due to the nature of dynamic change.

Previous studies showed that the MOT performance was typically impaired by objects’ dynamic changes in surface features (Makovski and Jiang, 2009; Zhou et al., 2010; Huff and Papenmeier, 2013; Meyerhoff et al., 2013, 2017; Papenmeier et al., 2014; Lyu et al., 2015). In a series of experiments conducted by Zhou et al. (2010), the tracking performance was significantly impaired when the topological properties of the moving objects changed. These findings suggested that feature changes might have a negative influence on identity verification and spatiotemporal continuity. Makovski and Jiang (2009) assumed that any objects’ features must remain continuous over time. Otherwise, when objects’ features changed and updated, it was insufficient for observers to take advantage of the objects’ uniqueness for improving tracking performance. They argued that objects’ surface features were retained in the VWM, and coding and maintaining the changed features occupied more attentional resources. Hence, the processing of unstable features would compete with attentive tracking for cognitive resources. Similar neuroimaging conclusions had been reached by comparing the brain activation in three feature swapping conditions (i.e., feature swapping within the target sets, feature swapping within the distractor sets, and feature un-swapping condition) during the attentive tracking (Lyu et al., 2015). Lyu et al. (2015) suggested that feature swapping within target sets increased the goal-driven attentional load. Specifically, the swapping enhanced the activation in the frontal eye field (FEF) and intraparietal sulcus (IPS). Overall, the change of surface features played a negative role in attentive tracking, and this negative effect might be related to the renewal of VWM. However, it remains unclear whether other factors would influence the effect of feature changes on attentive tracking.

Huff and Papenmeier (2013) indicated that tracking performance was impaired when the texture motion conflicted with the object motion. They showed that a short interval of approximately 100 ms was sufficient for observers to complete an integration of the texture motion and the object motion. Tracking performance would be impaired when the texture on the surface of an object moved in the opposite direction to the object’s movement. They suggested that the texture motion might have shifted the perceived object locations, thus affecting the tracking performance. Meyerhoff et al. (2013) in another study further indicated an object-specific tracking performance impairment for the targets with opposite texture motion, which suggested that the motion information was integrated in an object-based manner. According to the previous findings, the influence of feature changes on tracking performance appeared to be inconsistent; how the feature change would affect tracking performance might mainly depend on whether it affected the processing of spatiotemporal information. The surface feature information and the spatiotemporal information might be weighted differently. Papenmeier et al. (2014) provided evidence that surface feature information (i.e., distinct colors) can be used in tracking when spatiotemporal discontinuities were implemented by abrupt scene rotations, abrupt zooms, or a reduced presentation frame rate. However, when the spatiotemporal information was reliable, the surface feature information seemed not to affect tracking. Hein and Moore (2012) suggested a flexible-weighting view showing that the information used to establish the object correspondence was weighted according to the information availability. Papenmeier et al. (2014) applied this idea to tracking and proposed that the spatiotemporal information was highly reliable when it is continuous, so it should receive a relatively high weight. However, when the spatiotemporal information was less reliable, the surface features would receive a relatively high weight and be used in tracking.

A whole line of the above studies investigated the influence of the feature change on the local tracking performance. Furthermore, it implied that in the MOT tasks, the spatiotemporal information and the surface feature information were weighted according to their reliability and availability (Papenmeier et al., 2014). The present study intended to focus on the case of the surface feature change, aiming to explore whether there are situational differences in the surface feature utilization weight under this circumstance. Specifically, does the level of feature heterogeneity lead to differences in surface feature availability and therefore influence to what extent the identity features can be utilized for assisting tracking? To investigate this issue, we reviewed the literature related to feature changes and found that objects’ surface features in these studies differed from each other (Makovski and Jiang, 2009; Zhou et al., 2010; Huff and Papenmeier, 2013; Papenmeier et al., 2014). The high feature complexity of stimuli resulted in a great information load and a low capacity of VWM (Alvarez and Cavanagh, 2004; Liu et al., 2012). Hence, the heterogeneity between the target set and the distractor set might have a crucial influence on regulating to what extent the unstable surface features can be utilized in tracking. In addition, we also found that objects’ surface features in these studies changed with different frequencies (Makovski and Jiang, 2009; Zhou et al., 2010; Meyerhoff et al., 2013; Papenmeier et al., 2014). Frequent updating of the surface features would make it unable to register the feature information of the objects in VWM (Makovski and Jiang, 2009). Hence, the frequency of feature change seemed to play another significant role in regulating the effect of feature change on tracking performance. Moreover, the features were used repeatedly before and after feature change (Makovski and Jiang, 2009). Accordingly, the process of VWM updating might be confused by the recurring features (Papenmeier et al., 2014), which may make observers reduce the strategic use of features. Hence, researchers should avoid using repeated features as stimuli when manipulating feature changes, so the confusion caused by duplicate features in VWM could be eliminated.

In light of these findings, the present study aimed to explore two issues. One was when the features changed during motion, whether the feature heterogeneity between targets and distractors would alter the extent to which the observers would use feature information to assist tracking. The other was whether this moderating effect due to the feature heterogeneity remained constant as the frequency of feature change increased. In other words, these two questions focused on how the feature heterogeneity and feature-change frequency affecting the process of VWM updating during attentive tracking. It could hopefully provide some clarity to help reach a deeper understanding of the interactive mechanisms between the visual system and unstable features (or objects). Moreover, it could also provide evidence about how observers perform frequent updates about objects’ identities during attentive tracking. Accordingly, the present study can be regarded as a transitional simulation study to help with interpreting the situation, where features keep changing during an objects’ motion as in real-world scenarios.

## Experiment 1

Experiment 1 aimed to test whether the feature heterogeneity between targets and distractors could adjust the extent to which the feature information could be used in improving tracking performance. Essentially, we tried to examine how the feature heterogeneity between targets and distractors regulated the VWM updating of surface features. Four levels of feature heterogeneity (i.e., high level of feature heterogeneity, intermediate level of feature heterogeneity, low level of feature heterogeneity, and homogenous feature level/baseline level) were conducted. For comparing with dynamic changes in surface features (feature-changed condition), stable object features (feature-fixed condition) were set as the baseline to illustrate the damaging effect of feature changes on tracking performance. In this experiment, we hypothesized that (1) the surface feature instability would affect tracking performance and (2) the degree of changes in tracking performance would be regulated by the different levels of feature heterogeneity.

### Methods

#### Participants

A statistical power analysis was performed for sample size estimation. A 2 (feature consistency: feature-fixed or feature-changed) × 4 (feature heterogeneity: two-unique, four-unique, eight-unique, and homogeneous) within-participant’s design was examined in our experiment, so we considered the interaction as the main effect of the feature heterogeneity on difference scores between each feature consistency condition (i.e., the performance of feature-fixed condition minus that of feature-changed condition) to calculate the sample size. In other words, the estimation of sample size with our two-factorial within-participants design was converted to that with a one-factorial within-participants design. Accordingly, the configuration parameters in G^*^Power version 3.1 (Faul et al., 2007) were as following. The projected partial η^2^ of the interaction of this experiment was set at 0.25, considering to be stricter than the priori results from the similarly designed experiments of Makovski and Jiang (2009, Experiments 3a, b, the partial η^2^ of interaction was 0.71 and 0.40, respectively); the alpha level of two-tailed was set at 0.05; the power value was set at 0.95; the number of groups was set at 1; and the number of measurements was set at 4. Subsequently, it turned out that a sample size of 20 would have been required.

Based on this, we finally recruited 24 participants (17 females; average age: 21.17 ± 1.66 years), which was adequate for the main objective of this study. All participants reported normal or corrected-to-normal eyesight and normal color perception. All participants gave written consent before the experiment and received payment after the experiment. This study was approved by the Institutional Review Board and Ethics Committee.

#### Apparatus and Stimuli

The stimuli were displayed on a 17-inch CRT monitor with a resolution of 1,024 × 768 pixels and a refresh rate of 85 Hz. All displays were programmed in MATLAB 2012b^1^ using Psychophysics Toolbox (Brainard, 1997; Pelli, 1997). Participants were tested individually in a room with normal interior lighting. They viewed the monitor from a distance of approximately 57 cm, which made 1 cm on the screen subtend approximately 1 degree of visual angle.

**Figure 1:**
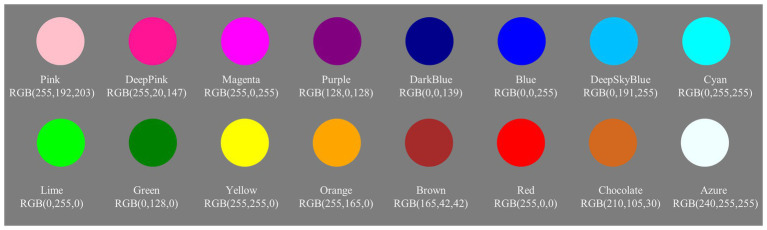
Samples of the stimulus used in Experiment 1.

We chose 16 colors as the possible surface features of the stimuli to ensure that no stimuli colors would be repeated before and after the change. The red, green, blue (RGB) space parameters of these colors were Pink [RGB (255, 192, 203)], Deep Pink [RGB (255, 20, 147)], Magenta [RGB (255, 0, 255)], Purple [RGB (128, 0, 128)], Deep Blue [RGB (0, 0, 139)], Blue [RGB (0, 0, 255)], Deep Sky Blue [RGB (0, 191, 255)], Cyan [RGB (0, 255, 255)], Lime [RGB (0, 255, 0)], Green [RGB (0, 128, 0)], Yellow [RGB (255, 255, 0)], Orange [RGB (255, 165, 0)], Brown [RGB (165, 42, 42)], Red [RGB (255, 0, 0)], Chocolate [RGB (210, 105, 30)], and Azure [RGB (240, 255, 255); see Figure 1]. These colors were chosen following the research of Makovski and Jiang (2009) and Wang et al. (2019), and then three psychology experts assessed the colors again to ensure color distinctiveness.

In each trial, a total of eight colored disks (0.625° radius) were used, and the color of each object depended on the specific conditions to which the current trial belonged. Half of the objects were flashed as targets by an outlined black circle [0.78° radius, 0.09° width, and RGB (0, 0, 0)]. All of them were confined to a centered rectangular area of 800 × 600 pixels (25° × 18.75°) with a black border [0.09° width, RGB (0, 0, 0)]. The background color was gray [RGB (128, 128, 128)] throughout the trial. At the beginning of each trial, the initial positions and moving directions of the disks were randomly chosen. The initial speed of the moving disks was set to 16°/s. During the motion, the movement speed of these disks varied randomly within the range of ±5% of the initial speed every 400 ms to avoid observers to predict the objects’ positions. The disks bounced off the edge of the rectangular border and repelled each other when they were tangent.

#### Design and Procedure

**Figure 2:**
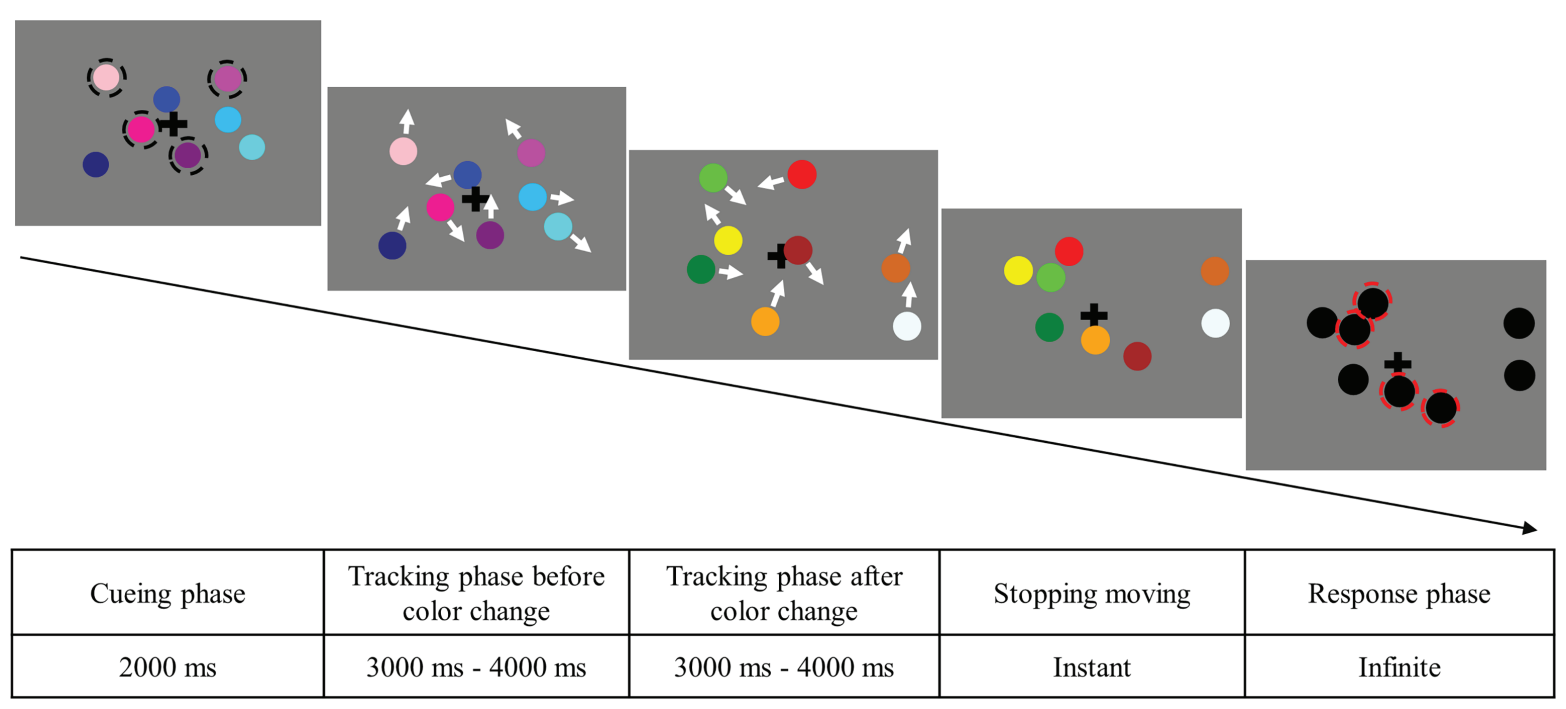
Sample illustration of a trial in the changed feature condition of Experiment 1.

All participants completed the experiment in an isolated experiment room. They were seated approximately 57 cm away from the monitor and were instructed to track four targets among a total of eight objects in each trial. At the beginning of each trial, eight objects were randomly assigned to nonoverlapping positions, and four targets were highlighted by black circles for 2,000 ms. Next, the black circles disappeared, and all objects moved randomly and independently in a nonoverlapping fashion within the presentation area. All objects changed their color halfway if it was in color-changed condition. The color of objects would not repeat before or after the color changes, while the color heterogeneity remained the same as before. All moving objects would end their movement and turn black at a varied time point between 6 and 8 s in each trial. When all objects stopped moving, the participants were required to pick four targets by clicking the mouse for an unlimited time. Moreover, they could guess in case they were uncertain. Once the participants selected four targets, they could press the spacebar to initiate the next trial (see Figure 2). Importantly, participants were informed that color was irrelevant and that their memory for colors would not be tested.

**Figure 3:**
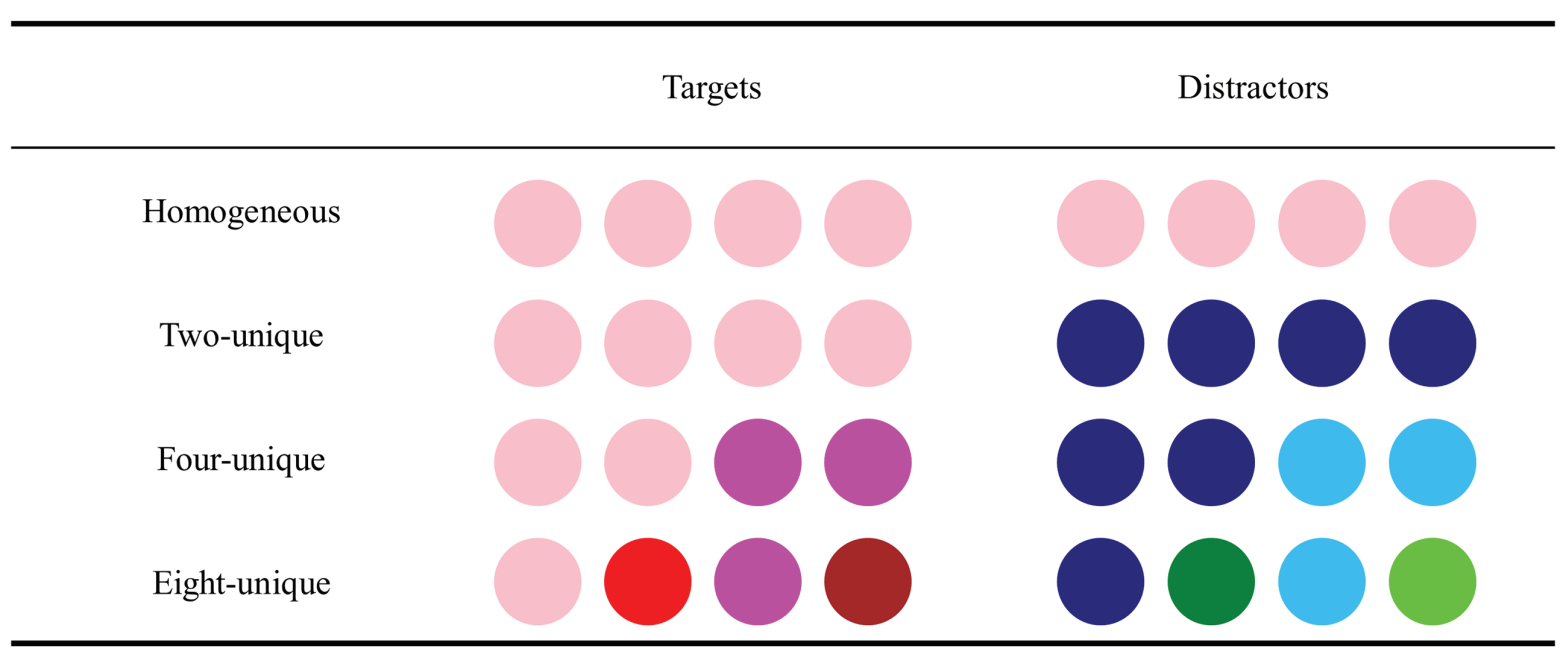
The four levels of the feature heterogeneity in Experiment 1.

We orthogonally manipulated the feature heterogeneity (two-unique, four-unique, eight-unique, and homogeneous; see Figure 3) and feature consistency (feature-fixed or feature-changed). In the feature-fixed condition, the colors of the objects were unchanged throughout the trial. In the feature-changed condition, the colors of all objects were changed once at the midpoint of the trial’s duration. In the two-unique condition, four targets shared a single color, and four distractors shared another color. In the four-unique condition, four targets were divided into two pairs, and each target pair independently shared one color, while four distractors were also divided into two pairs, and each distractor pair independently shared one color that was completely different from that of the target pair. In the eight-unique condition, all eight objects were different colors. In the homogeneous condition, all eight objects were identical in color, which was also regarded as the baseline level. The above four conditions (i.e., eight-unique, four-unique, two-unique, and homogeneous) in turn reflected the four hierarchical levels of feature heterogeneity (i.e., high level of feature heterogeneity, intermediate level of feature heterogeneity, low level of feature heterogeneity, and homogenous feature level/baseline level). After orthogonal treatment, the factors of feature heterogeneity and feature consistency could be combined into eight conditions. The color arrangement among the four heterogeneity conditions was still preserved after the color change, which ensured that the relationship of feature heterogeneity between objects was similar to its arrangement before the color changes. Furthermore, the color grouping between objects in each condition was retained regardless of the color change. For instance, in the four-unique condition, if two target objects shared the same color (e.g., both were red) before the color changes, then both of them would share another same color after the color changes (e.g., both of them turned to be green).

There were a total of 160 trials in this experiment, divided evenly into eight conditions (4 feature heterogeneity × 2 feature consistency; i.e., 20 trials in each condition). All trials were presented in a randomly intermixed order. In addition, participants needed to first complete eight practice trials to ensure that they understood the task before performing experimental trials. The dependent variable, tracking accuracy in both experiments was defined as the average proportion of correctly identified targets (Hulleman, 2005; Meyerhoff et al., 2017).

### Results and Discussion

**Figure 4:**
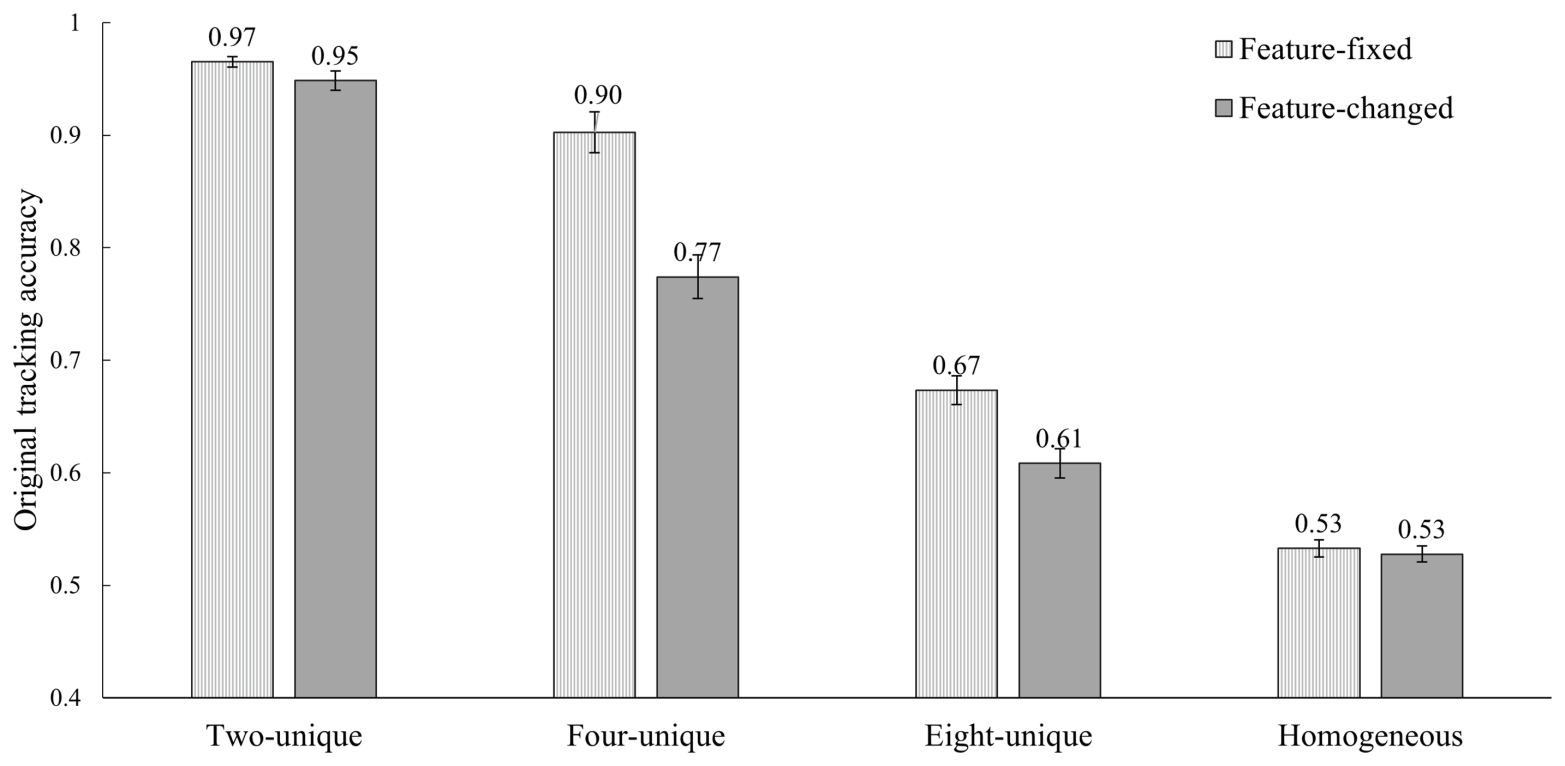
The tracking accuracies of the eight conditions in Experiment 1 (error bars show ±1 standard error of the mean).

The results of Experiment 1 are shown in Figure 4. The mean accuracies were submitted to a 4 (feature heterogeneity: two-unique, four-unique, eight-unique, and homogeneous) × 2 (feature consistency: the feature-fixed and feature-changed condition) within-participants repeated-measures ANOVA. The results revealed that the main effects of feature consistency [*F*(1, 23) = 67.00, *p* < 0.001, η^2^_p_ = 0.74] and feature heterogeneity [*F*(1.77, 40.68) = 475.70, *p* < 0.001, η^2^_p_ = 0.95, Greenhouse-Geisser corrected] on tracking accuracy were significant. Notably, the interaction effect between feature consistency and feature heterogeneity was also significant, *F*(3, 69) = 16.48, *p* < 0.001, η^2^_p_ = 0.42. This finding suggested that the effect of feature consistency on tracking performance could be significantly regulated by the different levels of feature heterogeneity.

Simple effect test (Bonferroni correction) was calculated to analyze the detailed differences among the four feature heterogeneity conditions in each feature consistency condition, and the detailed differences between the two feature consistency conditions in each feature heterogeneity condition. For the former, the results showed that both the eight-unique condition [*t*(23) = 4.34, *p* < 0.001, Cohen’s *d* = 0.82] and the four-unique condition [*t*(23) = 6.91, *p* < 0.001, Cohen’s *d* = 1.36] resulted in a significant impairment related to the feature change, while the two-unique condition [*t*(23) = 2.05, *p* = 0.052, Cohen’s *d* = 0.42] and the homogeneous condition [*t*(23) = 0.49, *p* = 0.625, Cohen’s *d* = 0.09] did not. It suggested that the change of feature did not always undermine tracking performance and the actual effect also depended on the level of feature heterogeneity. For the latter, not only in the feature-fixed condition [*F*(3, 21) = 977.21, *p* < 0.001, η^2^_p_ = 0.99], but also in the feature-changed condition [*F*(3, 21) = 605.15, *p* < 0.001, η^2^_p_ = 0.99], the tracking accuracy showed a significant downward trend, i.e., two-unique condition > four-unique condition > eight-unique condition > homogeneous condition, and the tracking accuracy of each condition in the front was significantly higher than that in the latter (*t*s > 3.60, *p*s < 0.01, Cohen’s ds > 0.74). Although feature changes did not change the relative hierarchical relationship between these conditions, the difference values between these conditions when feature-changed were to some extent different with that when feature-fixed. Thus, not only the uniqueness of stable features but also the uniqueness of unstable features could assist with attentive tracking. This finding also indicated that the effect of the feature heterogeneity was consistent in both stable/fixed and unstable/changed features, suggesting their processing mechanisms in VWM might partially overlap. In addition, except for the homogeneous condition, the trend of the other conditions could be interpreted that tracking accuracy decreased significantly as the increase of feature heterogeneity. Namely, the higher the feature heterogeneity, the lower the tracking performance.

To sum up, the results of Experiment 1 revealed that feature changes would impair attentive tracking in the four-unique and the eight-unique conditions rather than in the two-unique and the homogeneous conditions. Whether the features changed or not, the tracking accuracies of the three unique conditions (i.e., two-unique, four-unique, and eight-unique conditions) were significantly higher than those of homogeneous conditions. This implied that both the unstable features and stable features could be used to assist attentional tracking. Additionally, the extent that the observers would use the unstable and stable features in tracking was related to the level of feature heterogeneity. When the feature heterogeneity was at a relatively lower level, such as in the homogeneous and two-unique conditions, the unstable features did not significantly interfere with the tracking performance. On the contrary, when the feature heterogeneity was at a relatively higher level, such as in the four-unique and eight-unique conditions, the unstable features would significantly impair the tracking performance. Finally, whether the feature changed or not, the tracking accuracies among the three unique conditions formed a significant downward trend (i.e., two-unique > four-unique > eight-unique). In other words, except for the homogeneous condition, the tracking performance decreased as the increase of feature heterogeneity.

## Experiment 2

Experiment 1 provided evidence that feature heterogeneity could adjust the extent to which the observers would use unstable features for improving tracking performance. However, the results were achieved at a low frequency of feature change. In Experiment 2, we further investigated whether the feature heterogeneity had the same regulatory effect as the frequency of feature change increased.

### Methods

#### Participants

In this experiment, we derived the sample size based on the actual effect size of Experiment 1. The interaction effect of feature consistency and feature heterogeneity in Experiment 1 was 0.42 (η^2^_p_) beyond the large effect (Cohen, 1988). Thus, we were confident that a sample size of more than 20 participants might also be applicable to the current experimental design [i.e., 3 (change frequency) × 4 (feature heterogeneity)]. We stopped data collection on the day when we exceed this target sample of 20 participants. Finally, 22 undergraduate and graduate students (16 females; age: 20.82 ± 2.06 years) were recruited. With this sample size, we would have detected an effect size (η^2^_p_) of 0.24, which was also beyond the large effect. The other recruitment requirements and procedures were consistent with Experiment 1.

#### Apparatus and Stimuli

**Figure 5:**
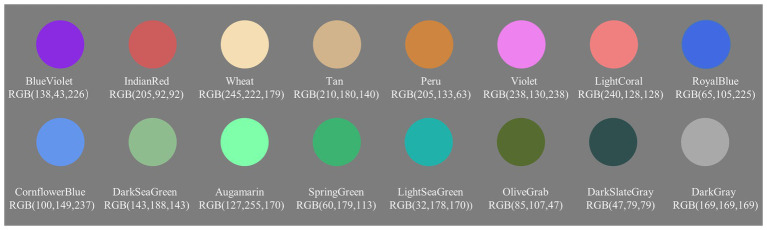
Samples of the added stimulus used in Experiment 2.

Except for the addition of 16 colors, the stimuli and apparatus in Experiment 2 were the same as in Experiment 1. An additional 16 colors were used to ensure that the color representations did not repeat before and after color change as the frequency of color change increased. The RGB space parameter of the added color was Blue Violet [RGB (138, 43, 226)], Indian Red [RGB (205, 92, 92)], Wheat [RGB (245, 222, 179)], Tan [RGB (210, 180, 140)], Peru [RGB (205, 133, 63)], Violet [RGB (238, 130, 238)], Light Coral [RGB (240, 128, 128)], Royal Blue [RGB (65, 105, 225)], Cornflower Blue [RGB (100, 149, 237)], DarkSea Green [RGB (143, 188, 143)], Aquamarine [RGB (127, 255, 170)], Spring Green [RGB (60, 179, 113)], LightSea Green [RGB (32, 178, 170)], Olive Grab [RGB (85, 107, 47)], DarkSlate Gray [RGB (47, 79, 79)], and Dark Gray [RGB (169, 169, 169); see Figure 5]. The initial motion speed of objects was randomly set to 14, 15, or 16°/s. During the motion in each trial, the speed of these disks would vary randomly within the range of ±5% of the initial speed every 400 ms (the same as in Experiment 1). In previous studies, differences in speed range appeared sometimes (Scholla et al., 2001; Suganuma and Yokosawa, 2006). Therefore, the speed range within 2°/s in the present study seems not to affect the current finding.

#### Design and Procedure

There were two factors, i.e., feature heterogeneity and feature-change frequency, to be investigated in this experiment, which were orthogonally combined to 12 conditions. The level of feature heterogeneity was divided into four conditions as in Experiment 1, namely, the two-unique, the four-unique, the eight-unique, and the homogeneous condition (baseline condition). The level of feature-change frequency was divided into three conditions, namely, the high, the intermediate, and the low frequency of feature changes. During attentive tracking, the high, the intermediate, and the low frequency of feature changes condition corresponded to three-changes, two-changes, and one-change of features, respectively.

Participants completed 240 experimental trials in total, divided randomly and evenly into the 12 conditions [4 (feature heterogeneity) × 3 (change frequency); i.e., 20 trials in each condition]. To avoid a fatigue effect in Experiment 2, all participants completed the task at two different sessions. In each session, the participants only needed to complete half (i.e., 10 trials) of each condition to avoid the time sequence effect. In addition, all participants were asked to finish eight trials as a practice to ensure they understood the task.

The procedure of Experiment 2 was fundamentally the same as in Experiment 1. The timing of the color change in each condition occurred with equal time intervals throughout the whole motion period. In one-change condition, all disks changed their color at the midpoint of the motion duration; in two-changes condition, the color change occurred when the motion duration passed 1/3 and 2/3; and in three-changes condition, the color change occurred when the duration of the entire movement has passed 1/4, 1/2, and 3/4.

### Results and Discussion

**Figure 6:**
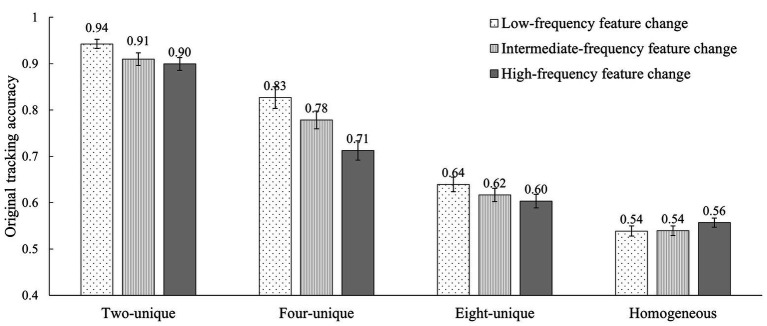
The tracking accuracies of the 12 conditions in Experiment 2 (error bars show ±1 standard error of the mean).

Figure 6 presents the average tracking performance as a function of the frequency of feature change in each feature heterogeneity condition. A 4 (feature heterogeneity: two-unique, four-unique, eight-unique, and homogeneous) × 3 (change frequency: three-changes, two-changes, and one-change) within-participants repeated-measures ANOVA showed the significant main effect on the frequency of feature change [*F*(2, 42) = 31.22, *p* < 0.001, η^2^_p_ = 0.60], and the level of feature heterogeneity [*F*(2.14, 44.88) = 299.61, *p* < 0.001, η^2^_p_ = 0.94, Greenhouse-Geisser correction], as well as the significant interaction effect between them [*F*(4.07, 85.37) = 6.49, *p* < 0.001, η^2^_p_ = 0.24, Greenhouse-Geisser correction]. These results indicated that the tracking performance would be affected by both the frequency of feature change and the level of feature heterogeneity. Furthermore, the effect of the frequency of feature change on tracking performance was adjusted by the different levels of feature heterogeneity.

The simple effect test was first applied to analyze the further effect of the frequency of feature change in each level of feature heterogeneity. The results showed that, the four-unique [*F*(2, 20) = 20.04, *p* < 0.001, η^2^_p_ = 0.67] and the two-unique [*F*(2, 20) = 6.78, *p* = 0.006, η^2^_p_ = 0.40] conditions showed a remarkably similar gradually decreasing trend, while the homogeneous condition [*F*(2, 20) = 2.54, *p* = 0.104, η^2^_p_ = 0.20] and the eight-unique condition [*F*(2, 20) = 2.91, *p* = 0.078, η^2^_p_ = 0.23] were not significantly different in each change frequency conditions. Furthermore, in the two-unique condition, the tracking accuracy of changing one time was significantly greater than those of changing two times [*t*(21) = 2.73, *p* = 0.038 (Bonferroni adjusted *p*-value, the same below), Cohen’s *d* = 0.58] and changing three times [*t*(21) = 2.70, *p* = 0.040, Cohen’s *d* = 0.57], but changing two times and changing three times were comparable [*t*(21) = 0.52, *p* > 0.99, Cohen’s *d* = 0.11]. In the four-unique condition, the tracking accuracy of changing one time was significantly greater than those of changing two times [*t*(21) = 3.75, *p* = 0.004, Cohen’s *d* = 0.81] and changing three times [*t*(21) = 6.16, *p* < 0.001, Cohen’s *d* = 1.31], and changing two times was also significantly greater than changing three times [*t*(21) = 5.89, *p* < 0.001, Cohen’s *d* = 1.26]. It was a clearly decreasing relationship between the frequency of feature changes and the tracking performance in two-unique and four-unique conditions rather than in homogeneous and eight-unique conditions. Moreover, this decreasing trend seemed to be more obvious as the feature-change frequency increased especially in four-unique conditions. Hence, the frequency of feature change could adjust to what extent the observers would use surface features on assisting tracking.

Next, another simple effect analysis (Bonferroni correction) was applied again to test whether the pattern of tracking performance difference caused by feature heterogeneity was similar in each frequency of feature change conditions. Similar to the findings of Experiment 1, the results of Experiment 2 also showed the significant downward trend in each of the feature-change frequency conditions, i.e., two-unique condition > four-unique condition > eight-unique condition > homogeneous condition [change one time: *F*(3, 19) = 261.02, *p* < 0.001, η^2^_p_ = 0.98; change two times: *F*(3, 19) = 169.29, *p* < 0.001, η^2^_p_ = 0.96; change three times: *F*(3, 19) = 176.42, *p* < 0.001, η^2^_p_ = 0.97], and the tracking accuracies of each condition in the front were significantly higher than that in the latter (*t*s > 2.96, *p*s < 0.05, Cohen’s ds > 0.63). Again, feature changes did not change the relative hierarchical relationship between these conditions in each feature-change frequency, although the difference values between these conditions were completely different in each feature-change frequency. In other words, feature-change frequency regulated the effect of feature heterogeneity on tracking performance to some extent. The frequency of feature change did not entirely interfere with the utilization of feature information in attentive tracking. Unstable features could be processed as well as stable features.

To sum up, the results of Experiment 2 nearly repeated those of Experiment 1. First, feature changes would impair attentive tracking. Second, tracking performance decreased as the increase of feature-change frequency in the two-unique and four-unique conditions rather than in the eight-unique and homogeneous conditions. Namely, feature heterogeneity regulated the effect of feature-change frequency on tracking performance. Third, except for the homogeneous condition, the higher the level of feature heterogeneity, the lower the tracking performance. The pattern of this trend was consistent under different frequencies of feature change and had great robustness. Fourth, the frequency of feature change did not entirely interfere with the utilization of feature information in attentive tracking. Unstable features as well as stable features could be used to assist attentive tracking.

## General Discussion

In real-world scenarios, objects’ surface features sometimes change as they move, impairing the object continuity. However, it remained unclear how our visual system adapted to this dynamic change. Thus, the present study investigated how humans performed in attentive tracking tasks when the features were frequently updated. Experiment 1 showed that feature changes could damage attentive tracking in the four-unique and the eight-unique conditions rather than in the homogeneous and the two-unique conditions. In addition, the tracking performance decreased as the increase of feature heterogeneity in the case of conditions with unique features (i.e., two-unique > four-unique > eight-unique). Thus, the feature heterogeneity regulated the damaging effect of feature change on tracking performance. Furthermore, Experiment 2 showed that there was a significant interaction between feature heterogeneity and feature-change frequency. The degree of tracking performance decline (related to change frequency) varied in each feature heterogeneity condition. Specifically, the tracking performance decreased significantly as the increase of feature-change frequency in the two-unique and the four-unique conditions rather than in the eight-unique and homogeneous conditions. Moreover, this decreasing trend became more obvious as the feature-change frequency increased especially in four-unique conditions. Similar to the results of Experiment 1, the tracking performance decreased significantly as the increase of feature heterogeneity when in the heterogeneous conditions (i.e., two-unique > four-unique > eight-unique). Moreover, the pattern of these downward trends (related to feature heterogeneity) was consistent at each frequency of feature change. In other words, although the difference in tracking performance between each feature heterogeneity condition was not exactly the same in each feature-change frequency, the feature-change frequency did not change the relative hierarchical relationship of the observers’ tracking performance in these conditions. In light of these findings, we concluded that unstable features could be utilized in attentive tracking as well as stable features, and the extent to which the observers rely on surface feature information to assist tracking depended on the level of feature heterogeneity and the frequency of feature change.

### The Effect of Feature Change on Tracking Performance

Observers’ tracking performance reflects the extent to which they maintain object continuity during the tracking (Zhou et al., 2010). The present results of feature change impairing the tracking performance support the idea that surface features play a role in maintaining object continuity (Makovski and Jiang, 2009; Papenmeier et al., 2014). It suggests that surface feature information might be simultaneously processed and stored in VWM during feature-changed tracking tasks. This is also in line with Wang et al. (2019) study that surface features of targets would be stored in VWM. When features changed during the motion, VWM constantly updated and restored the changed features, resulting in an increase of attentional demand. Thus, the higher the frequency of feature change was, the greater the attentional demand was used to maintain and update VWM. In addition, Makovski and Jiang (2009) claimed that the mechanism of utilizing surface feature information to maintain object continuity might be attributed to an effortful target recovery process. Feature change would lead to identity information unreliable and make the target recovery difficult. That is, this unreliability of feature information increased as the increase of feature-change frequency. Furthermore, Papenmeier et al. (2014) held the idea that when feature information became unreliable, the weight of spatiotemporal information arose and the target recovery functioned less. Thus, the inability to use identity information for target recovery would make the weight of feature information reduce and then degrade tracking performance. However, these hypotheses will need to be further verified through a dual-task paradigm in future studies.

### The Regulation Role of Feature Heterogeneity in the Effect of Feature-Change Frequency on Tracking Performance

Although the observers’ tracking performance was overwhelmingly impaired by the unstable features, our results revealed that the feature heterogeneity could regulate this negative effect. With the decrease of feature heterogeneity, the adverse effect related to feature changes could be alleviated or even eliminated. The current findings could be summarized in two aspects. One is the tracking performance of the heterogeneous features (i.e., the two-unique condition, the four-unique condition, and the eight-unique condition) was higher than that of the homogeneous features (i.e., the homogeneous/baseline condition). The other is under the conditions that object features being heterogeneous, the tracking performance increased as the level of feature heterogeneity decreased. These findings suggested that in the case of feature-changed conditions, to what extent the observers would use surface features in assisting tracking was related to the level of feature heterogeneity.

The mechanism of these findings might be interpreted from two aspects. On the one hand, the level of feature heterogeneity affected the targets recovery strategy. Compared with the homogeneous condition, tracking performance in conditions with the heterogeneous features showed a beneficial effect, providing support for the hypothesis that the heterogeneous features allowed observers to use the surface features for recovering lost targets (Makovski and Jiang, 2009). Moreover, the more complex the stimuli, the greater the information load and the lower the VWM capacity for stimuli features (Alvarez and Cavanagh, 2004; Liu et al., 2012). Accordingly, feature heterogeneity was closely related to feature complexity: the higher the feature heterogeneity, the higher the feature complexity. When feature changes occurred, the number of items needed to be updated in higher feature heterogeneity conditions was relatively larger, reflecting the higher level of working memory refresh load. The load of heterogeneous features processing, therefore, would increase as the level of feature heterogeneity increased. In other words, the higher feature heterogeneity made observers more difficult to utilize target recovery strategies and eventually failed to improve tracking performance.

On the other hand, the feature heterogeneity might influence the availability of the surface features. The flexible cognitive resource theory assumed that there was a trade-off between the number of items stored and the precision of each item (Franconeri et al., 2013). The lower feature heterogeneity would possibly allow individuals to reduce working memory updating load, and to allocate more attention resources to increase the number of updated items. Hence, the lower working memory load in the two-unique conditions and the four-unique conditions might reflect the higher availability of feature information. Surface feature information, therefore, would be weighted in higher priority and be used for assisting tracking in these conditions (Hein and Moore, 2012; Papenmeier et al., 2014). These two speculations would also need to be further tested.

In essence, how the feature heterogeneity regulated the effect of feature changing on tracking performance might be regarded as how the feature complexity regulated the function of VWM updating. The significant difference in tracking performance at different levels of heterogeneity suggested that individuals could use the surface feature information to assist attentive tracking, even when it changed or was unstable. It explained why the uniqueness of features could still contribute to attentional tracking during the VWM updating of unstable features.

In addition to feature heterogeneity, the efficiency of VWM updating also decreased with the increase of the frequency of feature changes under certain circumstances (i.e., two-unique condition and four-unique condition). Nevertheless, the moderation effect caused by the feature heterogeneity was not significantly interrupted by the feature changing frequency. Therefore, this downward trend of tracking performance caused by the feature changing frequency might be a systematic effect. Tracking performance seemed to be more affected by feature heterogeneity than by the frequency of feature changes.

### Connections to Previous Studies

Firstly, the current findings indicated that the uniqueness of both unstable and stable features could assist in attentive tracking, which was not consistent with the results of Makovski and Jiang (2009). They showed that the improved performance when tracking unique objects only appeared under the fixed-color condition rather than under the changing-color conditions. Two possible explanations may account for the discrepancy. The one was the difference in the frequency of feature change. In their experiments, it seemed like the slowest rate was 1,000 ms per change, while the fastest rate used in our experiments was ~1,500 ms per change. The higher the frequency of feature change, the more difficult for observers to consolidate visual information into VWM (Makovski and Jiang, 2009). The difference in the frequency of feature change indeed played a crucial role in the impact of uniqueness on tracking performance. The alternative was the confusion of VWM. In their experiments, the color information was adopted repeatedly in the process of feature updating, which might induce interference among memory retention, memory forgetting, and memory registration. Participants could not promptly and effectively refresh VWM and subsequently abandoned the use of color features. When this confusion was eliminated, the advantage of uniqueness occurred.

Besides, the current findings proved and expanded the study of Zhou et al. (2010). Their experimental settings were similar to the two-unique condition of the present study, in which targets shared one color and distractors shared another. Each item changed suddenly every 1–3 s with a limitation that they could not change simultaneously. Across a series of MOT tasks, their results showed that tracking performance was not disrupted when the moving objects underwent featural changes. However, it was significantly impaired when the objects changed the topological properties of their holes (Zhou et al., 2010). In the present study, the feature changes presentation was changed to the simultaneous display and mainly focused on changes in surface features rather than in topological properties. As a result, it verified that the tracking performance would also be impaired when the frequency of surface feature changes (i.e., color changes) increased.

Moreover, the current findings extended the understanding of how different forms of feature change affected tracking performance. Resemble studies examining another kind of feature change, namely texture, presented evidence that texture features could be utilized and integrated within 100 ms in an object-based manner (Huff and Papenmeier, 2013; Meyerhoff et al., 2013). Texture motion conflicting with object motion would impair tracking performance compared to consistent motion direction. The present study focused on color features and feature heterogeneity, presenting evidence that color features could be utilized even when surface features were unreliable. Furthermore, the extent to which surface features would be utilized in tracking varied in each feature heterogeneity condition. The above studies explored the influence of dynamic feature changes on attentive tracking with diverse perspectives, verifying the effects of specific forms of surface feature change on tracking performance and advancing the understanding of the role of dynamic surface features.

Finally, the current findings replenished evidence for a flexible-weighting view in attentive tracking (Hein and Moore, 2012; Papenmeier et al., 2014). Papenmeier et al. (2014) explored the role of feature information in conditions with spatiotemporal discontinuity. They found that spatiotemporal information particularly received a high weight when it was reliable. However, when it became unreliable, the weight of feature information arose. The current study further investigated the role of feature information when spatiotemporal information was reliable. The results suggested that when spatiotemporal continuity remains, the weight of feature information might also be flexible and be related to the level of feature heterogeneity.

In conclusion, the present research provided sufficient evidence that attentive tracking performance would be impaired by unstable surface features. Besides, the uniqueness of the surface features could also assist in attentive tracking when surface features changed. Furthermore, the weight of the surface feature utilization in assisting tracking was relatively related to the level of feature heterogeneity. Finally, the regulation effect was consistent in different frequencies of feature change. These findings suggested that the frequency of feature change interacted with feature heterogeneity. Taken together, exploring the role of unstable features in dynamic attentive tracking is a novel concept in the realm of cognitive processing of visual objects. It can help us to respond to the controversial question of what an object is from a different perspective. Future studies should make improvements through neuroimaging using the technique of functional magnetic resonance imaging (fMRI), event-related potentials (ERPs), and functional near-infrared spectroscopy (fNIRS).

## Data Availability Statement

The analyzed datasets of tracking accuracy for this study can be found in the OSF https://osf.io/mgau7/.

## Ethics Statement

The studies involving human participants were reviewed and approved by the Institutional Review Board and Ethics Committee of Human Participant Protection, Faculty of Psychology at Beijing Normal University. The patients/participants provided their written informed consent to participate in this study.

## Author Contributions

CZ and LH conceived and designed the experiments. CZ performed the experiments and analyzed the data. CZ, LH, LW, CW, XL, BH, and XZ contributed to the writing of the manuscript. All authors contributed to the article and approved the submitted version.

### Conflict of Interest

The authors declare that the research was conducted in the absence of any commercial or financial relationships that could be construed as a potential conflict of interest.

## Footnotes

^1^http://www.mathworks.com
